# A Rare Case of Aggressive Non-Hodgkin's Lymphoma of the Muscle in a Young Male Presenting as Necrotizing Myofasciitis

**DOI:** 10.7759/cureus.59888

**Published:** 2024-05-08

**Authors:** Vaishali Mehta, Javed Iqbal, Jagadish Akella, Khawaja Zaki

**Affiliations:** 1 Pulmonary and Critical Care Medicine, Nassau University Medical Center, East Meadow, USA

**Keywords:** non-hodgkin’s lymphoma, rhabdomyolysis, compartment syndrome, chemotherapy, mechanical ventilation, critical care, skeletal muscle, iris lymphoma, hiv/aids, necrotizing myofasciitis

## Abstract

Non-Hodgkin's lymphoma (NHL) involving skeletal muscle is generally found to be a secondary metastasis and extremely rarely as a primary site of malignancy. Furthermore, in HIV patients, an increased incidence of lymphomas may be identified within the first six months of highly active antiretroviral therapy (HAART) initiation unmasked by immune reconstitution inflammatory syndrome (IRIS). We illustrate an extremely rare instance of NHL of the skeletal muscle in a young immunocompromised male with HIV/AIDS presenting as necrotizing myofasciitis complicated by compartment syndrome and hemodialysis-refractory type B lactic acidosis.

A young Hispanic male with AIDS was admitted for acute left thigh pain and was soon found to have abscess formation with compartment syndrome requiring thigh fasciotomy. During the course of the ICU stay, the patient’s clinical status acutely worsened with sepsis-induced multiorgan failure, including acute renal and acute liver failure requiring N-acetylcysteine and severe refractory metabolic acidosis requiring renal replacement therapy. Repeat imaging demonstrated diffuse myonecrosis. Left thigh muscle biopsy confirmed aggressive NHL of skeletal muscle. Despite months of arduous medical management in ICU, doxorubicin, vincristine, cyclophosphamide chemotherapy with concurrent high-dose prednisone for the vented patient, and intermittent curves of improvement, our patient succumbed to the nature of the disease and subsequently died from severe sepsis from the immunocompromised state.

Interestingly, our patient’s initial CD4 count was 1, which improved to 96 after five months of HAART, raising concerns for IRIS lymphoma. Given such rapid improvement with chemotherapy, the possibility of IRIS-related lymphoma, and the surprising dearth of data for chemotherapy use in critically ill patients on mechanical ventilation, more research is needed in these topics to better approach such complicated patients.

## Introduction

Non-Hodgkin's lymphoma (NHL) involving skeletal muscle is generally found to be a secondary metastasis. It is extremely rare for such a manifestation to be a primary site of malignancy [1]. Most patients with this unique illness are usually over 50 years old and have a clearly defined mass seen on imaging [1-4]. However, our case documents its manifestation in a young male, presenting instead with diffusely hypertrophied muscle in the context of compartment syndrome with rhabdomyolysis and refractory type B lactic acidosis.

Primary NHL of skeletal muscle groups is extremely rare and has been reported in males above 50 years. It accounts for only 1.4% of total cases of all lymphomas, with 1.1% being NHL. Furthermore, primary skeletal muscle NHLs are even more sporadically documented, comprising 1.5% of the total NHL cases [1-4]. In skeletal muscle NHL, the tumor and its swelling spreads from one compartment to another leading to compartment syndrome, significant morbidity and mortality with life-threatening rhabdomyolysis, and multiorgan failure. Interestingly, in HIV patients, an increased incidence of lymphomas may be identified within the first six months of highly active antiretroviral therapy (HAART) initiation unmasked by immune reconstitution inflammatory syndrome (IRIS). Our patient’s initial CD4 count was 1, which improved to 96 after five months of HAART, raising concerns for IRIS lymphoma [5].

## Case presentation

A Hispanic male in his 30s with a past medical history of COVID-19-related pericardial effusion and human immunodeficiency virus (HIV) with admission CD4 of 53 cells/uL (on bictegravir, emtricitabine and tenofovir alafenamide, and prophylactic trimethoprim-sulfamethoxazole) was admitted for acute left thigh pain for past two to three weeks. The patient denied any trauma to the area. Pertinent surgical history included a pericardial window for pericardial effusion diagnosed earlier in the year with no residual complications. Social history was negative for alcohol, smoking, or drug use, with no pertinent family history. On exam, the patient was hemodynamically stable with tachycardia to 110 and significant physical exam findings revealed a left thigh that was swollen, tender, erythematous, and warm to touch.

**Figure 1:**
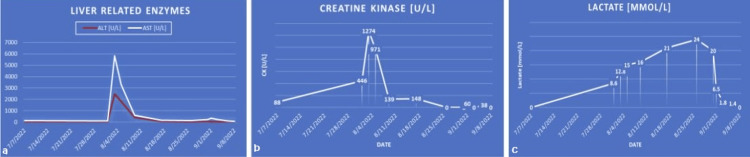
Graphic depiction of labs discussed in Table 1 (a) Improvement of liver-related enzymes was seen after administration of N-acetylcysteine. (b) Improvement in rhabdomyolysis was seen after the start of continuous renal replacement therapy (CRRT). (c) Improvement in lactic acidosis was seen after the administration of chemotherapy.

CT of the left lower extremity with contrast initially showed diffuse heterogeneous expansion of quadricep muscles in the left thigh containing multiple irregular collections of fluid. Labs on admission were grossly within normal limits, as described below in Table 1. With worsening pain over the next four days, repeat CT revealed an interval increase in diffuse heterogeneous expansion of thigh muscles with abscess formation requiring CT-guided left lower extremity deep soft tissue drainage by interventional radiology in which 2 ml of brown viscous fluid was taken and sent to the lab for further analysis. Two days after the drainage, the patient was noted to have worsening swelling in the left lower extremity and tense compartments prompting class I thigh compartment fasciotomy. The patient remained intubated and sedated after surgery with clinical deterioration. The patient continued to spike a fever of 103°F. A pan-CT scan was performed, which showed a bilateral lower lobe lung consolidative process and opacification of bilateral temporal mastoid air cells with mucosal thickening of bilateral sphenoidal sinuses and opacification of the right frontal sinus and he was treated with broad-spectrum antibiotics vancomycin and Zosyn; however, over the course of next two weeks, the patient later developed distributive shock with multiorgan failure, including acute renal failure, acute liver failure, and severe metabolic acidosis requiring hemodialysis (Figure 1 and Table 1).

**Table 1:** Hospital course and lab trends Lab results and trends from the day of baseline/admission to the end of 1st cycle of chemotherapy. Lab results were consistent with severe metabolic acidosis, rhabdomyolysis, acute renal failure, and acute liver failure. Notes dictate the importance of the date and labs reported. Acute liver failure resolved with N-acetylcysteine (NAC) administration and lactic acidosis and acute renal failure requiring continuous renal replacement therapy (CRRT) improved after the administration of chemotherapy once the patient was diagnosed via muscle biopsy. MICU: medical intensive care unit; Hgb: hemoglobin; PLT: platelets; ALT: alanine aminotransferase; AST: aspartate aminotransferase; CK: creatine kinase; LDH: lactate dehydrogenase.

	3/16/22	7/7/2022	8/1/2022	8/3/2022	8/5/2022	8/9/2022	8/17/2022	8/26/2022	8/31/2022	9/1/2022	9/3/2022	9/6/2022	9/8/2022
Notes	Baseline during HIV clinic visit	Day 1: Admission to hospital	Day 24: Compartment syndrome	Day 26: MICU consult	Day 28: After 1 round of NAC treatment & day 1 of CRRT	Day 32: 3 rounds of NAC & day 4 of CRRT	Day 40: After 12 days of CRRT	Day 49: After chemotherapy day 1	Day 54: Chemo day 5	Day 55: Round 2 of chemo	Day 57: CRRT stopped, 3 days after completion of chemo	Day 60: 6 days after completion of chemo	Day 62: 8 days after completion of chemo
WBC (4.5-11 K/mm3)	5	7	13.02	10.3	11.4	16.88	16.49	7.23	14.31	6.05	3.16	1.75	0.65
Hgb (13.5-18 g/dL)	7.8	8.8	8.2	7.3	8.7	8.3	8.8	7.6	9	7.3	8.2	8.4	9
PLT (150-450 K/mm3)	804	654	277	363	311	221	220	117	150	83	77	110	148
Bicarb (20-31 mmol/L)	25	22	13	16	14	22	16	22	12	26	27	27	18
Cr (0.7-1.3 mg/dL)	0.5	0.6	0.8	3.9	4.9	2.6	1.2	0.5	0.5	0.5	0.6	0.6	0.5
ALT (7-40 U/L)	39	16	17	2492	1814	410	79	52	59	45	54	45	40
AST (13-40 U/L)	32	105	89	3342	1480	190	77	95	172	295	182	56	23
CK (46-171 U/L)	102	88	446	1274	971	139	148	NA/-	NA/-	60	NA/-	38	NA/-
Lactate (0.5-1.6 mmol/L)	1.3	NA/-	8.6	12.8	15	16	21	24	20	6.5	1.8	1.4	NA/-
LDH (140-280 U/L)	NA/-	NA/-	>3840	>3840	NA/-	>3840	2136	1737	3750	NA/-	>3840	>3840	NA/-
CD4 (500-1500 cells/uL)	<20	53	NA/-		NA/-		20	26	NA/-	NA/-	NA/-	NA/-	NA/-

**Figure 2:**
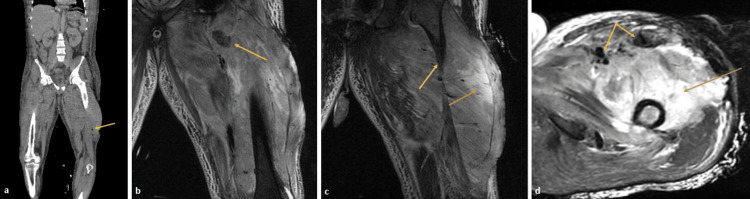
Imaging consistent with myofasciitis and diffuse myositis (a) Small pockets of air seen in distal posterior thigh (yellow arrow) on coronal view of CT left lower extremity. (b) MRI showing a focal area with low T2 and high T1 signal intensity within the iliopsoas muscle measuring approximately 2.2 x 1.9 x 2.8 cm suspected to reflect an area of focal myonecrosis (yellow arrow). (c) Diffuse subcutaneous soft tissue edema (orange arrow) with small ill-defined fluid collections (yellow arrow) within the deep subcutaneous soft tissues adjacent to the fasciotomy site. (d) Diffuse subcutaneous soft tissue edema (orange arrow) with small ill-defined fluid collections (yellow arrow) within the deep subcutaneous soft tissues adjacent to the fasciotomy site.

Repeat CT demonstrated findings concerning necrotizing fasciitis and prompted MRI, which demonstrated a diffuse myonecrosis considered likely sequelae of compartment syndrome or deep tissue inflammatory process such as myositis (Figure 2). His further workup, including transthoracic echocardiogram, showed trace pericardial effusion, hyperdynamic global left ventricular systolic function with ejection fraction of 70%, and normal right ventricular size and function. Blood cultures remained negative.

Due to refractory lactic acidosis and persistent finding of diffuse tissue edema and inflammation of quadricep muscle on repeat CT, with negative rheumatologic workup, including myositis and vasculitis panel with aldolase, anti-Jo, antineutrophil cytoplasmic antibody (ANCA), scl-70, and anticentromere antibodies, other causes of type B lactic acidosis, including oncological etiologies, were considered. Surgical re-exploration and muscle biopsy were performed.

**Figure 3:**
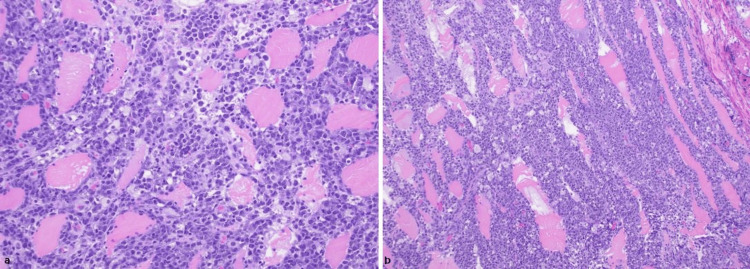
Left thigh muscle biopsy (a) Left thigh biopsy showed extensive infiltration of pleomorphic lymphocytes with irregular vesicular nuclei and scant cytoplasm. (b) The image shows scattered apoptotic cells, mitotic figures, and macrophages that were interspersed imparting a "starry sky" appearance.

Left thigh biopsy showed extensive infiltration of large-sized pleomorphic lymphocytes with irregular vesicular nuclei, variably prominent nucleoli, and scant cytoplasm. They also showed scattered apoptotic cells, mitotic figures, and tingible body macrophages that were seen interspersed, imparting a "starry sky" appearance. Further immunohistochemistry analysis was positive for CD20, CD79a, CD10, and BCL6, and negative for BCL2, CD5, and TdT, with Ki67 +90%. Fluorescence in situ hybridization (FISH) was positive for MYC and negative for BCL2 and BCL6 as well as negative for Epstein-Barr virus (EBV) in-situ hybridization. These results were consistent with aggressive NHL diffuse large B-cell lymphoma of skeletal muscle (Figure 3).

The oncology team was consulted and a decision was made to start the patient on chemotherapy. The patient tolerated the first cycle of doxorubicin, vincristine, cyclophosphamide, and high-dose prednisone (CHOP therapy); however, rituximab was held in light of low CD4 count (<20).

Subsequently, over the week, a marked improvement was noted in the patient’s status with rapid normalization of lactate (24+ → 20 → 6.5 → 4.2 → 1.8 → 1.4). The patient was also slowly weaned off sedation drips of fentanyl, midazolam, and dexmedetomidine and started on spontaneous breathing trials. Due to prolonged ICU stay and critical illness myopathy and delirium, the patient failed the weaning trial and subsequently underwent tracheostomy and percutaneous endoscopic gastrostomy (PEG) tube placement. However, the patient subsequently died of sepsis due to a severe immunocompromised state.

## Discussion

In our review of the literature pertaining to this case, we noted that lymphomatous involvement in muscle groups had been rarely reported. It accounted for only 1.4% of total cases, with a further division of about 0.3% of those being Hodgkin's lymphoma and 1.1% being NHL. Furthermore, the primary skeletal muscle non-Hodgkin's cases are sporadic even among NHL cases, with these only comprising about 1.5% of the total NHL cases. Additionally, most of these cases have been reported in males above 50 years [1-4]. This makes our patient’s case of a primary skeletal muscle NHL in a 35-year-old an exceedingly rare occurrence.

Muscle involvement in lymphoma may occur in three different ways: (1) as part of metastatic disease, (2) as an extension of malignancy from adjacent bone or lymph nodes, and lastly, (3) as a primary site of muscle lymphoma [6]. This pathology affects the lower extremities most commonly (88%). It is 60 times more evident in the elderly and immunocompromised patients, including non-compliant HIV patients with low CD4 counts (<100 cells/mL). This makes a case for considering this type of lymphoma with primary site involvement as a part of our differential diagnoses in such patients, especially when dealing with rapidly deteriorating or refractory cases.

In skeletal muscle NHL, the tumor and its swelling may spread from one compartment to other adjacent compartments, sometimes leading to compartment syndrome, severe electrolyte disorders, acidosis, and rhabdomyolysis similar to what was seen in our patient, but our case is unique as it is found in a young patient with HIV [7]. Diagnosis can usually be made via an MRI or a PET scan, which shows a diffuse enlargement of the involved muscle and adjacent organs and increased metabolisms at the muscle level. However, in most cases, like ours, the gold standard for definitive diagnosis remains surgical biopsy of the tumor. Some cases may show adjacent bony abnormalities with bone destruction if it spreads to bones [8].

Randomized trials have been performed to address the treatment of patients with diffuse large B-cell lymphoma (DLBCL) with CHOP and R-CHOP (rituximab, cyclophosphamide, doxorubicin, vincristine, and prednisone). Evaluation for maintenance rituximab (MR) vs. observation was performed by Habermann et al., which showed, in untreated DLBCL patients, three-year failure-free survival (FFS) for R-CHOP was 53% and 43% for CHOP (p = 0.04) with reduced risk of death seen with R-CHOP (p = 0.05). Rituximab maintenance after CHOP showed prolonged FFS (p = 0.0004) but not after R-CHOP therapy (p = 0.81). This indicated that rituximab administration, given during induction or maintenance with CHOP therapy, significantly prolonged FFS within DLBCL patients [9]. Though definitive therapy was to begin R-CHOP, the oncology team only started CHOP and held rituximab as it must be used cautiously in patients with CD4 < 50 cells/uL, given the high risk for developing a fatal infection, but benefits may outweigh the risks if CD4 is higher [10].

In HIV patients, an increased incidence of lymphomas may be identified within the first six months of antiretroviral therapy initiation. This might be attributed to the unmasking effect caused by IRIS. IRIS is characterized by paradoxical worsening of an infectious or inflammatory process in light of increasing CD4 count as a result of treatment (CD4 average at 173 cells/uL; interquartile range = 73-302 cells/uL). Gopal et al. studied lymphoma IRIS in a cohort study that revealed that out of 482 lymphoma patients, 12% of the patients met the criteria for IRIS and had increased mortality compared to non-IRIS lymphoma [5]. Upon retrospective analysis of our patient's chart, we found that the patient’s initial CD4 count upon HIV diagnosis was <20 cell/uL, and after treatment with bictegravir, emtricitabine, and tenofovir alafenamide for five months, it had increased up to 96 cells/uL prior to admission, raising concern for the possibility of IRIS related worsening of lymphoma symptoms. Though uncommon, this should also be considered as a part of the differential diagnoses for HIV patients who have initiated HAART in the previous six months.

## Conclusions

Most patients with this unique illness are usually over 50 years old and have a defined mass on imaging. However, our case documents this malignancy’s manifestation in a young male, presenting as a left leg abscess clinically with diffusely hypertrophied muscle progressing to necrotizing myofasciitis, compartment syndrome with severe rhabdomyolysis, and refractory type B lactic acidosis. The rapid clinical improvement with chemotherapy seen in our patient makes a case for the need for more research into the relationship between lymphomas in immunocompromised states, especially in the setting of IRIS, unmasking as well as instigating more research on the role of chemotherapy in critically ill patients on mechanical ventilation.
